# Mendelian randomization suggests a causal relationship between gut dysbiosis and thyroid cancer

**DOI:** 10.3389/fcimb.2023.1298443

**Published:** 2023-12-01

**Authors:** Feng Zhu, Pengpeng Zhang, Ying Liu, Chongchan Bao, Dong Qian, Chaoqun Ma, Hua Li, Ting Yu

**Affiliations:** ^1^ Department of Gastroenterology, The First Affiliated Hospital with Nanjing Medical University, Nanjing, China; ^2^ Department of Gastroenterology, The First People’s Hospital of Kunshan, Suzhou, China; ^3^ Department of Lung Cancer Surgery, Tianjin Medical University Cancer Institute and Hospital, Tianjin, China; ^4^ Department of Thoracic Surgery, The First Affiliated Hospital with Nanjing Medical University, Nanjing, China; ^5^ Department of General Surgery, Affiliated Hospital of Nanjing University of TCM, Jiangsu Province Hospital of TCM, Nanjing, China; ^6^ Department of Breast and Thyroid Surgery, The Affiliated Hospital of Youjiang Medical University for Nationalities, Baise, China; ^7^ Department of General Surgery, The Affiliated Hospital of Youjiang Medical University for Nationalities, Baise, China

**Keywords:** Mendelian randomization, bidirectional, causal relationship, gut dysbiosis, thyroid cancer

## Abstract

**Background:**

Alterations in gut microbiota composition and function have been linked to the development and progression of thyroid cancer (TC). However, the exact nature of the causal relationship between them remains uncertain.

**Methods:**

A bidirectional two-sample Mendelian randomization (TSMR) analysis was conducted to assess the causal connection between gut microbiota (18,340 individuals) and TC (6,699 cases combined with 1,613,655 controls) using data from a genome-wide association study (GWAS). The primary analysis used the inverse-variance weighted (IVW) method to estimate the causal effect, with supplementary approaches including the weighted median, weighted mode, simple mode, and MR-Egger. Heterogeneity and pleiotropy were assessed using the Cochrane Q test, MR-Egger intercept test, and MR-PRESSO global test. A reverse TSMR analysis was performed to explore reverse causality.

**Results:**

This study identified seven microbial taxa with significant associations with TC. Specifically, the genus *Butyrivibrio* (OR: 1.127, 95% CI: 1.008-1.260, *p* = 0.036)*, Fusicatenibacter* (OR: 1.313, 95% CI: 1.066-1.618, *p* = 0.011)*, Oscillospira* (OR: 1.240, 95% CI: 1.001-1.536, *p* = 0.049)*, Ruminococcus2* (OR: 1.408, 95% CI: 1.158-1.711, *p* < 0.001)*, Terrisporobacter* (OR: 1.241, 95% CI: 1.018-1.513, *p* = 0.032) were identified as risk factors for TC, while The genus *Olsenella* (OR: 0.882, 95% CI: 0.787-0.989, *p* = 0.031) and *Ruminococcaceae UCG004* (OR: 0.719, 95% CI: 0.566-0.914, *p* = 0.007) were associated with reduced TC risk. The reverse MR analysis found no evidence of reverse causality and suggested that TC may lead to increased levels of the genus *Holdemanella* (β: 0.053, 95% CI: 0.012~0.094, *p* = 0.011) and decreased levels of the order *Bacillales* (β: -0.075, 95% CI: -0.143~-0.006, *p* = 0.033). No significant bias, heterogeneity, or pleiotropy was detected in this study.

**Conclusion:**

This study suggests a potential causal relationship between gut microbiota and TC, providing new insights into the role of gut microbiota in TC. Further research is needed to explore the underlying biological mechanisms.

## 1 Introduction

Thyroid cancer (TC) is the most common form of endocrine malignant neoplasm, with steadily increasing global incidence over recent decades (Kitahara and Sosa, 2016; Lin et al., 2021; Siegel et al., 2023). It is projected to become the fourth most prevalent cancer worldwide by 2030 (Kim et al., 2020). TC, while generally having a favorable survival rate, imposes significant healthcare costs and long-term quality of life challenges for patients (Lubitz et al., 2014; Hedman et al., 2016; Wang et al., 2018). As a result, TC has become a global health concern.

The exact pathogenesis of TC remains unclear, but it is widely accepted that genetic and environmental factors play significant roles in its development (Kim et al., 2020). The maintenance of a healthy gut microbiota composition is crucial for overall health, particularly for the immune and endocrine systems (Li et al., 2019). Recent research has highlighted the intricate connection between gut microbiota and the thyroid gland, often referred to as the gut-thyroid axis (Su et al., 2020; Sagaram et al., 2022; Biscarini et al., 2023; Cao et al., 2023). Dysbiosis, or an imbalance in gut microbiota composition and function, has been implicated in the etiology and progression of thyroid disorders, including TC (Zhang et al., 2019; Samimi and Haghpanah, 2020; Li et al., 2021; Ishaq et al., 2022; Lu et al., 2022). However, studies investigating the link between gut microbiota and TC have produced inconsistent findings, and the precise role of gut microbiota in TC development remains uncertain. It is unclear whether they play a causal role or are simply a consequence of shared risk factors.

Mendelian randomization (MR) is a novel statistical approach that allows researchers to assess causal relationships in potential exposure-outcome pathways. MR uses genetic variants as instrumental variables to act as proxies for exposure phenotypes, making it less susceptible to the influence of environmental confounders and reverse causality (Xu et al., 2021). While MR has been extensively used to study the relationship between gut microbiota and thyroid disorders, limited evidence exists regarding the causal effects of gut microbiota on TC. This study conducted a comprehensive two-sample Mendelian randomization (TSMR) analysis to investigate the potential causal connection between gut microbiota and TC, providing insights into the role of gut microbiota in TC pathogenesis and potential therapeutic interventions.

## 2 Materials and methods

### 2.1 Study design

**Figure 1:**
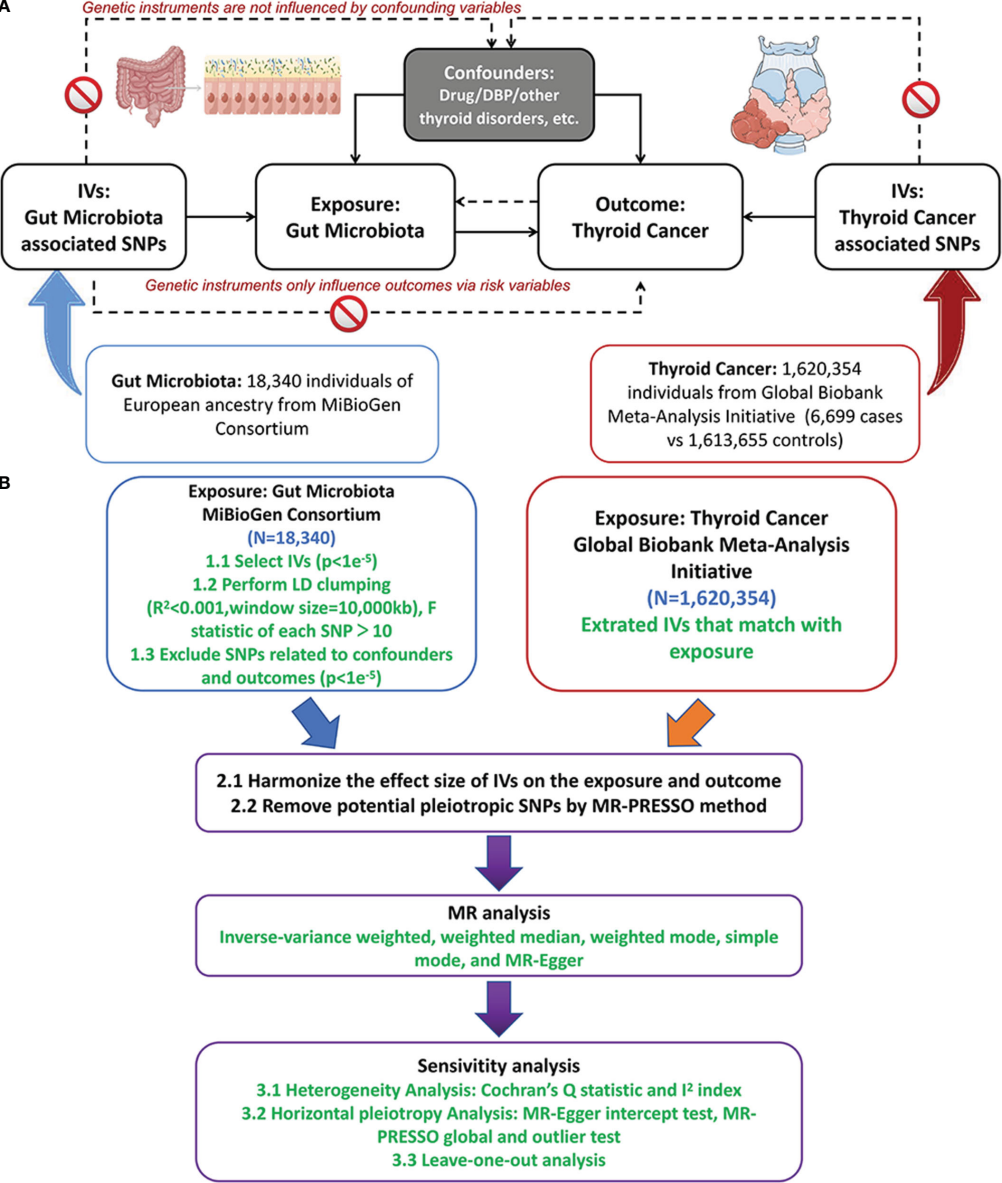
The study design of MR analysis **(A)** and the overall workflow **(B)**. MR, mendelian randomization;DBP, diastolic blood pressure; IVs, instrument variables; LD, linkage disequilibrium; SNP, single nucleotide polymorphism; MR-PRESSO, MR pleiotropy residual sum and outlier. Figure 1 was produced using Figdraw (https://www.figdraw.com/static/index.html).

This study employed a TSMR approach to investigate the causal relationship between gut microbiota and thyroid cancer across multiple taxonomic levels, including 9 phyla, 16 classes, 20 orders, 35 families, and 131 genera. **Figure 1** depicted the study design and illustrated the essential MR assumptions (Bowden et al., 2015; Sanderson et al., 2019): (1) the IVs should exhibit a strong and consistent association with gut microbiota, (2) IVs should not be associated with any confounding factors, and (3) IVs solely influenced thyroid cancer via the pathway of the exposure factors.

### 2.2 Data sources

Genetic data on gut microbiota were obtained from a comprehensive meta-analysis of genome-wide association studies (GWAS) involving 18,340 individuals from 24 cohorts, conducted by the MiBioGen consortium (www.mibiogen.org) (Kurilshikov et al., 2021). A total of 211 bacterial taxa and 122,110 associated single nucleotide polymorphisms (SNPs) were identified, with 15 bacterial taxa lacking specific species names excluded. GWAS meta-analyses on thyroid cancer were sourced from the Global Biobank Meta-Analysis Initiative (GBMI), which included 6,699 cases and 1,613,655 controls (Zhou et al., 2022). Ethical approval was obtained for the use of the original data.

### 2.3 The selection of instrumental variables

Firstly, to enhance the number of available SNPs, a lenient threshold of p < 1 × e^-5^ was employed (Long et al., 2023; Yu et al., 2023). Similarly, in the reverse analysis investigating the impact of thyroid cancer on the gut microbiota, IVs were chosen with a loose cutoff of p < 5 × e^-8^. Subsequently, linkage disequilibrium (LD) was employed to evaluate the existence of gene linkage among these SNPs, employing an *r^2^* value of 0.001 and a window size of 10,000 kb. The formula F = beta^2^_exposure_/se^2^_exposure_ and R^2 =^ 2×(1-MAF)×MAF×beta^2^ were utilized to assess the statistical significance of SNPs, and the SNPs with weak associations (F < 10) were eliminated (Xie et al., 2023). During the coordination process of the exposure and outcome data, incompatible and palindromic SNPs were eliminated. Furthermore, potential pleiotropic confounders were identified using the Phenoscanner database (http://www.phenoscanner.medschl.cam.ac.uk/) (Kamat et al., 2019). The findings of our investigation indicated that eight SNPs exhibited associations with potential risk factors for thyroid cancer. Specifically, rs17379710, rs113379006 and rs10931481 were found to be linked with “Treatment with levothyroxine sodium” or “Self-reported hypothyroidism or myxoedema,” while rs12124567, rs1035691, rs6489992, rs17708276, and rs113379006 demonstrated an association with diastolic blood pressure (Huang et al., 2022), which were all excluded from the current study.

### 2.4 MR analysis

In this study, we utilized a bidirectional TSMR analysis to explore the causal connection between the gut microbiome and thyroid cancer, employing four common MR methods: inverse-variance-weighted (IVW), weighted median (WM), MR-Egger regression, and Mendelian randomization pleiotropy residual sum and outlier (MR-PRESSO). Additionally, we employed the Wald ratio test to examine features exclusively encompassing one IV (Burgess et al., 2017). The IVW approach served as the primary method for estimating the causal effect, aiming to ascertain the cumulative impact of all SNPs (Burgess et al., 2013). The WM method is endowed with the capacity to integrate information from multiple genetic variants, yielding a singular causal estimation, provided that at least fifty percent of the weight originates from valid IVs (Bowden et al., 2016). The MR-Egger method, grounded on the assumption of InSIDE, can yield unbiased estimates of causal relationships, even in scenarios where all instrumental SNPs are invalidated due to pleiotropy (Bowden et al., 2015). It is worth noting that if these methods yield inconsistent outcomes, we will accord priority to IVW as the principal result.

Furthermore, we conducted a set of sensitivity analyses to ensure the validity and dependability of our findings. We applied the MR-PRESSO global test and the MR Egger intercept test to evaluate the presence of global horizontal pleiotropy in the IVs. A p-value surpassing 0.05 would indicate the absence of statistically significant horizontal pleiotropy (Verbanck et al., 2018). In this study, we employed Cochran’s Q statistic (MR-IVW) to detect heterogeneity, where a p-value exceeding 0.05 signified the absence of heterogeneity (Hemani et al., 2018). Simultaneously, we performed a leave-one-out sensitivity analysis on the significant results to ascertain whether a single SNP was accountable for the observed causal relationship in the TSMR analysis (Kurilshikov et al., 2021). Additionally, we executed the reverse TSMR procedure in accordance with the previously mentioned TSMR analysis. The study design adhered to the STROBE-MR (Strengthening the Reporting of Observational studies in Epidemiology - Mendelian randomization) guideline (Skrivankova et al., 2021).

### 2.5 Statistical analysis

For a more stringent interpretation of the causal link, the Bonferroni-adjusted significance criterion was applied, considering the feature level of each bacteria: genera: 0.05/119 (4.2 × 10−4), families: 0.05/32 (1.56 × 10−3), orders: 0.05/20 (2.5 × 10−3), classes: 0.05/16 (3.1 × 10−3), and phyla: 0.05/9 (5.5 × 10−3) (Shi et al., 2023). Microbiomes exhibiting *p* values below 0.05/n were identified as having a notably likely connection with thyroid cancer, while those falling within the range of 0.05 to the adjusted value were considered to have a nominal causal influence (Georgakis et al., 2019; Yu et al., 2021; Long et al., 2023). All computations were carried out employing TwoSampleMR (version 0.5.6) and R version 4.3.0 (R Foundation for Statistical Computing).

## 3 Findings

### 3.1 The influences of gut microbiota on TC patients

Upon excluding SNPs linked to potential TC risk factors, a total of 1,479 IVs were included in the examination of gut microbiota’s causal effects on TC (**Supplementary Table 1**). The F-statistics for all IVs exceeded 10, indicating the absence of weak instrument bias (**Supplementary Table 2**).

**Figure 2:**
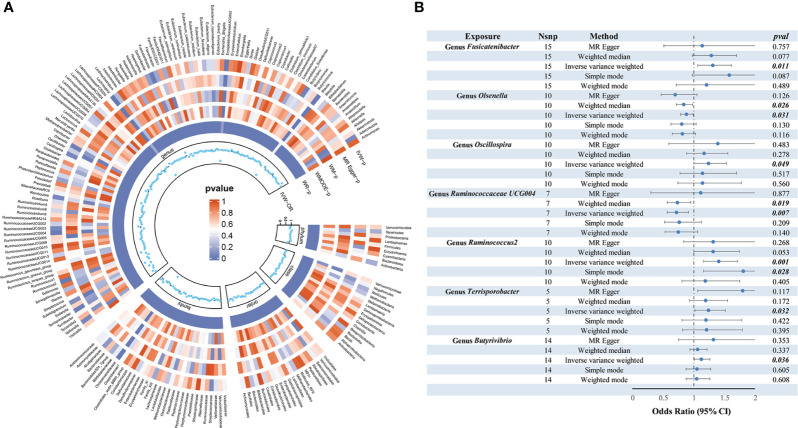
MR analysis of gut microbiota on thyroid cancer. **(A)** From outside to inside, the P values of IVW, MR Egger, WM, WMODE, and WR are represented, respectively. **(B)** Forest plots of MR results for seven gut microbiota taxa on thyroid cancer. MR, Mendelian Randomization; NSNP, number of SNPs; IVW, inverse-variance weighted; WM, weighted median; WMODE, weighted mode; WR, Wald Ratio; OR, odds ratio.

**Figure 3:**
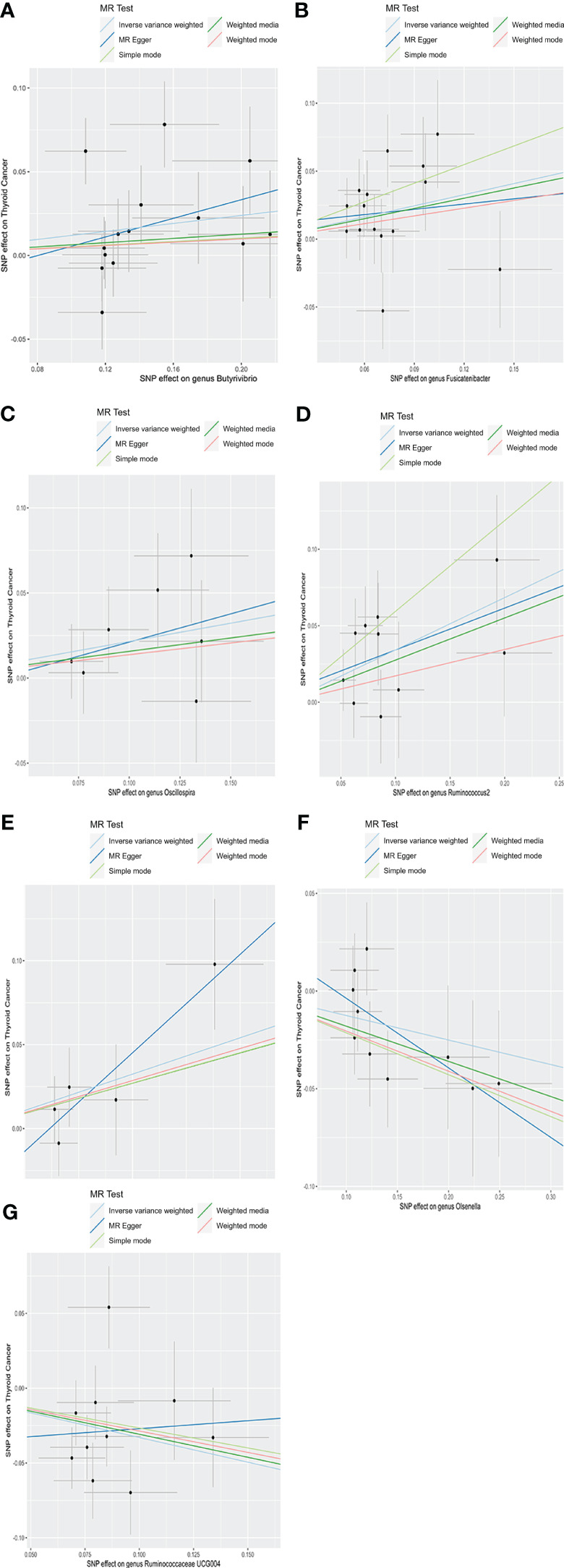
Scatter plots of causal estimates of exposure (specific gut microbiota) on thyroid cancer. **(A)** *Genus Butyrivibrio*; **(B)** *Genus Fusicatenibacter*; **(C)** *Genus Oscillospira*; **(D)** *Genus Ruminococcus2*; **(E)** *Genus Terrisporobacter*; **(F)** *Genus Olsenella*; **(G)** *Genus Ruminococcaceae UCG004*. MR, Mendelian Randomization.

As depicted in **Figure 2**, employing the IVW method, it was revealed that a genetically projected higher abundance of the genus *Butyrivibrio* (OR: 1.127, 95% CI: 1.008-1.260, *p* = 0.036), *Fusicatenibacter* (OR: 1.313, 95% CI: 1.066-1.618, p = 0.011), *Oscillospira* (OR: 1.240, 95% CI: 1.001-1.536, p = 0.049), *Ruminococcus2* (OR: 1.408, 95% CI: 1.158-1.711, p < 0.001), *Terrisporobacter* (OR: 1.241, 95% CI: 1.018-1.513, p = 0.032) was linked to an increased TC risk. Conversely, heightened genetically projected levels of the genus *Olsenella* (OR: 0.882, 95% CI: 0.787-0.989, p = 0.031) and *Ruminococcaceae UCG004* (OR: 0.719, 95% CI: 0.566-0.914, p = 0.007) were associated with a decreased risk of TC. **Figure 3** illustrates the scatterplot depicting the gut microbiome’s causal effect on TC.

The sensitivity analyses in this study did not yield any indications of heterogeneity, as revealed by Cochrane’s Q test of the IVW method (**Table 1**). Additionally, employing the leave-one-out method did not pinpoint any instances where a single SNP significantly impacted the observed outcomes across the seven positive findings (**Supplementary Figure 1**). Furthermore, as shown in **Table 1**, the MR-Egger intercept test and MR PRESSO global test affirmed the absence of horizontal pleiotropy.

**Table 1:** Results of heterogeneity and horizontal pleiotropy for the effect of gut microbiota on thyroid cancer.

Exposure	Cochrane Q statistic	MR Egger intercept	MR Egger intercept *pval*	MR PRESSO Global Test *pval*	Outliers
Genus *Fusicatenibacter*	0.297	0.010	0.722	0.453	NA
Genus *Olsenella*	0.657	0.032	0.283	0.328	NA
Genus *Oscillospira*	0.702	-0.012	0.794	0.669	NA
Genus *Ruminococcaceae* UCG004	0.069	-0.038	0.525	0.718	NA
Genus *Ruminococcus2*	0.618	0.007	0.751	0.109	NA
Genus *Terrisporobacter*	0.459	-0.043	0.233	0.659	NA
Genus *Butyrivibrio*	0.075	-0.022	0.584	0.098	NA

MR, mendelian randomization.

### 3.2 Reverse analysis of TSMR: influence of TC on gut microbiota

**Figure 4:**
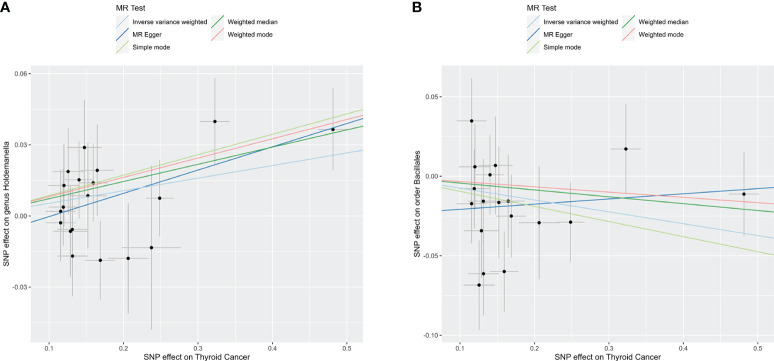
Reverse TSMR analysis. Scatter plots of causal estimates of exposure (thyroid cancer) on specific gut microbiota. **(A)** *Genus Holdemanella*; **(B)** *Order Bacillales*. MR, Mendelian Randomization.

To explore the potential causative influence of TC on gut microbiota, 19 TC-associated SNPs from the GBMI were utilized as instrumental variables (IVs) (**Supplementary Table 5**). Utilizing the IVW method, an elevation in the abundance of genus *Holdemanella* was observed following the onset of TC, while TC was linked to a reduction in the levels of order *Bacillales* (**Table 2**). **Figure 4** illustrates the scatterplot depicting the causal estimates of TC exposure on specific gut microbiota. Furthermore, it was ascertained that the IVs were devoid of any indications of weak instrument bias or heterogeneity statistics, and no evidence of horizontal pleiotropy was identified between the IVs and the gut microbiota (**Supplementary Table 6**). Leave-one-out analysis indicated that none of the SNPs significantly impacted the MR outcomes (**Supplementary Figure 2**).

**Table 2:** MR results of causal effects for TC on gut microbiota.

Exposure	Outcome	Method	Nsnp	Beta	SE	95% CI	*pval*
Thyroid Cancer	Genus *Holdemanella*	MR Egger	19	0.098	0.045	0.009-0.187	**0.046**
		Weighted median	19	0.073	0.030	0.013 -0.132	**0.017**
		Inverse variance weighted	19	0.053	0.021	0.012-0.094	**0.011**
		Simple mode	19	0.086	0.051	-0.015-0.187	0.111
		Weighted mode	19	0.081	0.031	0.021-0.142	**0.017**
	Order *Bacillales*	MR Egger	18	0.033	0.072	-0.108-0.173	0.656
		Weighted median	18	-0.043	0.044	-0.130-0.044	0.332
		Inverse variance weighted	18	-0.075	0.035	-0.143~-0.006	**0.033**
		Simple mode	18	-0.095	0.083	-0.257-0.068	0.268
		Weighted mode	18	-0.033	0.049	-0.130-0.063	0.508

SNP, single nucleotide polymorphism; MR, mendelian randomization; SE, standard error; CI, confidence interval.

Bold values represented P<0.05.

## 4 Discussion

To the best of our knowledge, a thorough and comprehensive exploration of the links between gut microbiota and TC, utilizing publicly accessible genetic data, has been conducted for the first time. The results of this investigation indicate that, at the level of genera, an increase in the abundance of *Butyrivibrio, Fusicatenibacter, Oscillospira, Ruminococcus2*, and *Terrisporobacter* may contribute to an elevated risk of TC, while heightened levels of *Olsenella* and *Ruminococcaceae UCG004* are associated with a reduced TC risk. In the subsequent reverse MR analysis, it was observed that the onset of TC might lead to a decrease in the abundance of genus *Holdemanella* and an increase in the level of order *Bacillales*.

Gut dysbiosis is a common phenomenon observed in individuals with thyroid disorders. Also, it has been the subject of recent research providing compelling evidence of the crucial interplay between gut microbiota and the thyroid gland, thus suggesting the presence of a robust gut-thyroid axis (Lerner et al., 2017; Liu et al., 2022). It has been reported that the gut microbiota and its metabolites have the potential to influence thyroid hormone homeostasis by triggering immune-inflammatory responses, modifying iodothyronine metabolism, and affecting the absorption of thyroid-associated micronutrients (Jiang et al., 2022). Multiple studies have been conducted to probe into the relationship between gut microbiota and thyroid diseases, including TC. Feng et al. noted that TC patients exhibit a higher level of gut microbiota richness and diversity (α-diversity) compared to a control group of healthy individuals (Feng et al., 2019). Moreover, a predictive model comprising eight genera was employed to distinguish the TC status, encompassing *Bacteroides, Lactococcus, Blautia, Eubacterium_coprostanoligene_group, Christensenellaceae_R-7_group, Ruminococcus_gnavus_group, Eubacerium_hallii_group*, and *Lactobacillus* (Feng et al., 2019). Similarly, Ishaq HM et al. also indicated significant intestinal bacterial overgrowth in TC patients (Ishaq et al., 2022). However, the study conducted by Lu et al. revealed a marked decrease in the diversity and richness of the gut microbiota in TC patients, with core genera closely linked to lipid metabolism, namely *Christensenellaceae_R-7_group* and *Eubacterium_coprostanoligenes_group* (Lu et al., 2022). In an exploratory cohort involving 90 TC patients and 90 healthy controls, Yu et al. also demonstrated a significant decrease in the richness and diversity of gut microbiota in TC patients (Yu et al., 2022). In their study, TC patients with metastatic lymphadenopathy exhibited a microbial signature characterized by elevated levels of genus *Fusobacterium* and *Alistipes*, alongside reduced levels of *Hungatella* and *Phascolarctobacterium* (Yu et al., 2022).

Despite the varying findings, it is noteworthy that these studies have reported a decline in the abundance of bacteria producing short-chain fatty acids (SCFAs) in TC patients (Jiang et al., 2022), indicating that the reduction of SCFAs-producing bacteria may contribute to the progression of TC (Yu et al., 2022). In this study, the genus *Ruminococcaceae UCG004* (OR: 0.719), a bacterium producing SCFAs, was identified as a protective factor against TC. It has been reported that SCFAs have the ability to inhibit the activity of histone deacetylase, serving as epigenetic modifiers that disrupt the expression of sodium/iodide symporter (NIS) and iodine uptake in TC cells (Samimi and Haghpanah, 2020; Jiang et al., 2022). Thus, SCFAs-producing bacteria like *Bifidobacterium* and *Faecalibacterium prausnitzii* have been employed in TC therapy (Lin et al., 2022; Fernandes et al., 2023; Zheng et al., 2023). However, other SCFAs-producing bacteria, including *Butyrivibrio* (OR: 1.127), *Fusicatenibacter* (OR: 1.313), *Oscillospira* (OR: 1.240), and *Ruminococcus2* (OR: 1.408), were identified as pathogenic factors for TC, offering a new perspective for future research. Additionally, the genus *Terrisporobacter* (OR: 1.241), a Gram-positive bacterium belonging to the family *Peptostreptococcaceae*, has also been implicated as a causative agent of TC in the current investigation, with documented potential to cause infections in human beings, especially in postoperative individuals with comorbidities (Wang et al., 2023). However, research on the correlation between the genus *Terrisporobacter* and TC is scarce, necessitating further exploration.

Recently, a considerable body of research has been dedicated to examine the influence of gut dysbiosis on immune responses in cancer (Wang et al., 2020). Genus *Olsenella* (OR: 0.882) and *Ruminococcaceae UCG004* (OR: 0.719), identified as “favorable” intestinal bacteria against TC in this study, have demonstrated potential in the field of tumor immunotherapy (Gopalakrishnan et al., 2018; Mager et al., 2020). According to Gopalakrishnan et al., patients with high diversity and abundance of *Ruminococcaceae/Faecalibacterium* exhibited enhanced systemic and anti-tumor immune responses through heightened antigen presentation and improved effector T cell function in both the periphery and the tumor microenvironment (Gopalakrishnan et al., 2018). Furthermore, it has been reported that the genus *Olsenella*, in combination with *Bifidobacterium pseudolongum* and *Lactobacillus johnsonii*, significantly improved the effectiveness of immune checkpoint inhibitors in murine cancer models (Mager et al., 2020). Thus, the present study may offer a fresh perspective on TC immunotherapy, particularly for advanced TC (Laha et al., 2020), from the standpoint of gut microbiota. Overall, limited research has documented the association between these intestinal bacteria and TC, providing new avenues for future studies. Hence, further studies are warranted to explore the specific mechanism of these intestinal bacteria in TC.

This study introduces several innovative elements. Firstly, it adopts a bidirectional TSMR approach based on comprehensive GWAS data to investigate the relationship between gut microbiota and TC, which can mitigate the influence of confounding variables and reverse causation frequently encountered in conventional observational analyses. Moreover, stringent screening criteria for instrumental variables (IVs) were applied, ensuring the absence of heterogeneity or pleiotropy in the outcomes, thereby affirming the robustness of our findings. Subsequently, the analysis on the causal relationship between specific gut microbiota and TC was carried out at various taxonomic levels, ranging from genus to phylum, potentially offering valuable insights for the clinical management of TC. Lastly, our research provides genetic evidence supporting the existence of the gut-thyroid axis, thus emphasizing the strong correlation between gut microbiota and TC.

Nonetheless, it is crucial to acknowledge the limitations of our study. Firstly, the GWAS summary data utilized predominantly consisted of patients of European heritage, potentially leading to biased estimates and limited applicability to diverse ethnic groups. Secondly, our findings did not meet the strict Bonferroni correction. However, it is important to note that the Bonferroni correction is a conservative approach that may result in false negatives (Li et al., 2013; Gupta et al., 2014). Finally, the absence of demographic data in the primary study, such as gender, age, and race, hindered the execution of additional subgroup analyses aimed at attaining more precise correlations.

## 5 Conclusion

Through a bidirectional TSMR approach, our study reveals that the genera *Butyrivibrio, Fusicatenibacter, Oscillospira, Ruminococcus2*, and *Terrisporobacter* may contribute to an increased risk of TC, while elevated levels of *Olsenella* and *Ruminococcaceae UCG004* are associated with a lower TC risk. The abundance of these specific gut microbiota may potentially serve as novel indicators for TC, and the manipulation of gut microbiota could potentially serve as a viable approach for the management and prevention of TC.

## Data availability statement

The original contributions presented in the study are included in the article/**Supplementary Material**. Further inquiries can be directed to the corresponding authors.

## Author contributions

FZ: Conceptualization, Data curation, Investigation, Methodology, Writing – original draft, Writing – review & editing. PZ: Conceptualization, Data curation, Investigation, Writing – original draft, Writing – review & editing. YL: Conceptualization, Investigation, Software, Writing – review & editing. CB: Writing – review & editing, Software. DQ: Conceptualization, Writing – original draft. CM: Investigation, Software, Writing – review & editing. HL: Writing – review & editing, Validation, Visualization. TY: Conceptualization, Data curation, Investigation, Methodology, Software, Writing – original draft, Writing – review & editing.
