# Supplementary material for: Mendelian randomization suggests a causal relationship between gut dysbiosis and thyroid cancer
Source: Front Cell Infect Microbiol. 2023 Dec 1;13:1298443. doi: 10.3389/fcimb.2023.1298443 (PMC10722196; doi:10.3389/fcimb.2023.1298443)
Supplement: Supplementary file 2 [file DataSheet_1.docx]

Supplementary Material

**Mendelian Randomization Suggests a Causal Relationship Between Gut Dysbiosis and Thyroid Cancer**

**Feng Zhu^1,2†^, Pengpeng Zhang^3,4†^, Ying Liu^5†^, Chongchan Bao^6†^, Dong Qian^5^, Chaoqun Ma^5^, Hua Li^7*^, Ting Yu^1*^**

***Correspondence to:**

Ting Yu, [njmuyt@163.com](mailto:njmuyt@163.com)

Hua Li, lihua_gx@ymun.edu.cn


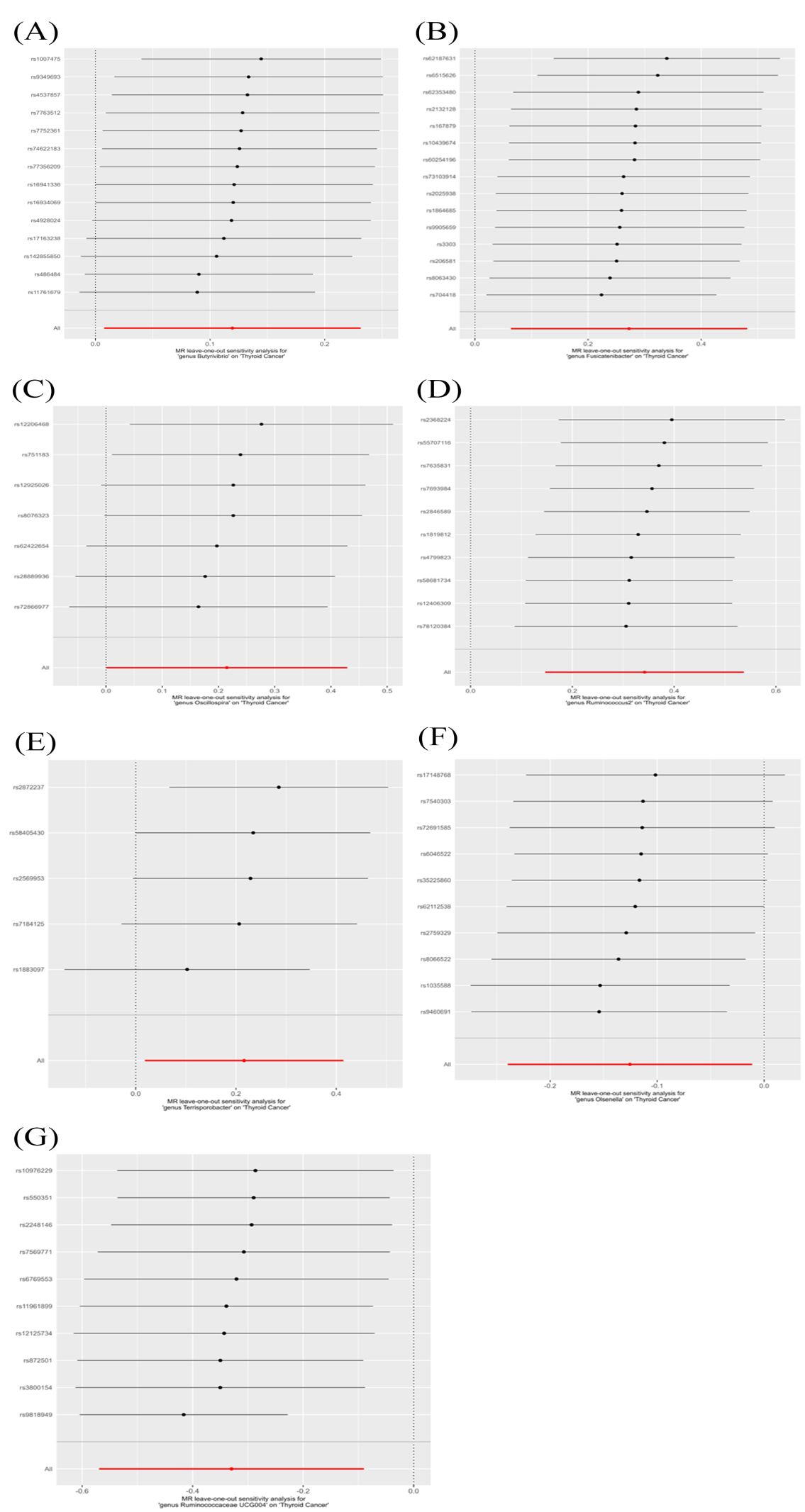


**Supplementary Figure S1.** Leave-one-out stability tests causal estimates of exposure (specific gut microbiota) on thyroid cancer. (A) *Genus Butyrivibrio*; (B) *Genus Fusicatenibacter*; (C) *Genus Oscillospira*; (D) *Genus Ruminococcus2*; (E) *Genus Terrisporobacter*; (F) *Genus Olsenella*; (G) *Genus Ruminococcaceae UCG004*.

**
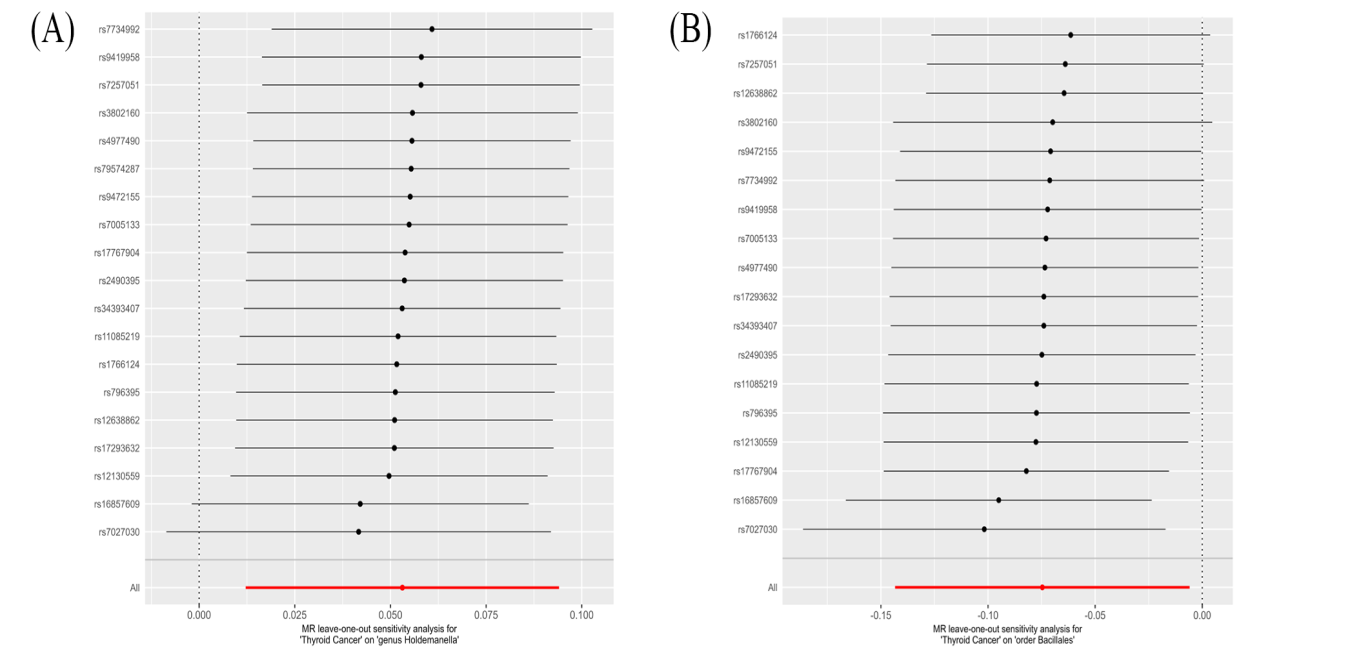
**

**Supplementary Figure S2.** Leave-one-out stability tests causal estimates of exposure (thyroid cancer) on specific gut microbiota. (A) *Genus Holdemanella*; (B) *Order Bacillales*.
